# A stochastic simulation of skeletal muscle calcium transients in a structurally realistic sarcomere model using MCell

**DOI:** 10.1371/journal.pcbi.1006712

**Published:** 2019-03-07

**Authors:** Robert John Holash, Brian R. MacIntosh

**Affiliations:** Human Performance Laboratory, Faculty of Kinesiology, University of Calgary, Calgary, Alberta, Canada; University of Washington, UNITED STATES

## Abstract

Skeletal muscle contraction is initiated when an action potential triggers the release of Ca^2+^ into the sarcomere in a process referred to as excitation-contraction coupling. The speed and scale of this process makes direct observation very challenging and invasive. To determine how the concentration of Ca^2+^ changes within the myofibril during a single activation, several simulation models have been developed. These models follow a common pattern; divide the half sarcomere into a series of compartments, then use ordinary differential equations to solve reactions occurring within and between the compartments. To further develop this type of simulation, we have created a realistic structural model of a skeletal muscle myofibrillar half-sarcomere using MCell software that incorporates the myofilament lattice structure. Using this simulation model, we were successful in reproducing the averaged calcium transient during a single activation consistent with both the experimental and previous simulation results. In addition, our simulation demonstrated that the inclusion of the myofilament lattice within our model produced an asymmetric distribution of Ca^2+^, with more Ca^2+^ accumulating near the Z-disk and less Ca^2+^ reaching the m-line. This asymmetric distribution of Ca^2+^ is also apparent when we examine how the Ca^2+^ are bound to the troponin-C proteins along the actin filaments. Our simulation model also allowed us to produce advanced visualizations of this process, including two simulation animations, allowing us to view Ca^2+^ release, diffusion, binding and uptake within the myofibrillar half-sarcomere.

## Author summary

In this study we develop a structural stochastic diffusion model, to study how calcium ions diffuse and interact within a skeletal muscle sarcomere following a simulated muscle activation. This type of model allows us to explore how structural elements, namely the actin and myosin filaments, within the sarcomere affect calcium diffusion, and the model provides a level of resolution for ligand-protein reactions that is currently unattainable with other types of diffusion models. This type of model is novel in that we topologically represent common protein structures within the sarcomere model. This topological representation of the myofibril includes the actin and myosin proteins which compose the filament lattice, the shape of the sarcoplasmic reticulum (SR), the placement of the SR bound calcium pumps (SERCA), as well as the position of the calcium release sites (RYR receptors) within the triad (SR and T-tubule connection), which together describe the micro-architecture of the sarcomere. This type of modelling is also unique as it allows the results to emerge from the interaction of the defined parameters powered by the random nature of diffusion. Using this new model we are able to show that calcium ions do not diffuse in a uniform fashion from the calcium release site, instead the diffusion is biased towards z-disks where there are fewer myosin filaments. We also see evidence that previous modelling techniques may be underestimating both the release rate from troponin-c, and the transfer rate of SERCA.

## Introduction

Skeletal muscle has a complex multi-scale structure. A single muscle cell contains over 2000 myofibrils arranged in parallel. The long thin myofibrils contain the contractile protein filaments, actin and myosin. The actin and myosin filaments are arranged in individual repeating units called sarcomeres, which are linked end-to-end in series. The sarcomere is the basic contractile unit within skeletal muscle and exhibits mirror symmetry about the middle. As such the sarcomere has two functional and independent halves. Muscle contraction is initiated when an action potential triggers calcium ions (Ca^2+^) to be released from the sarcoplasmic reticulum (SR), into the myofibrillar sarcoplasm.

**Fig 1:**
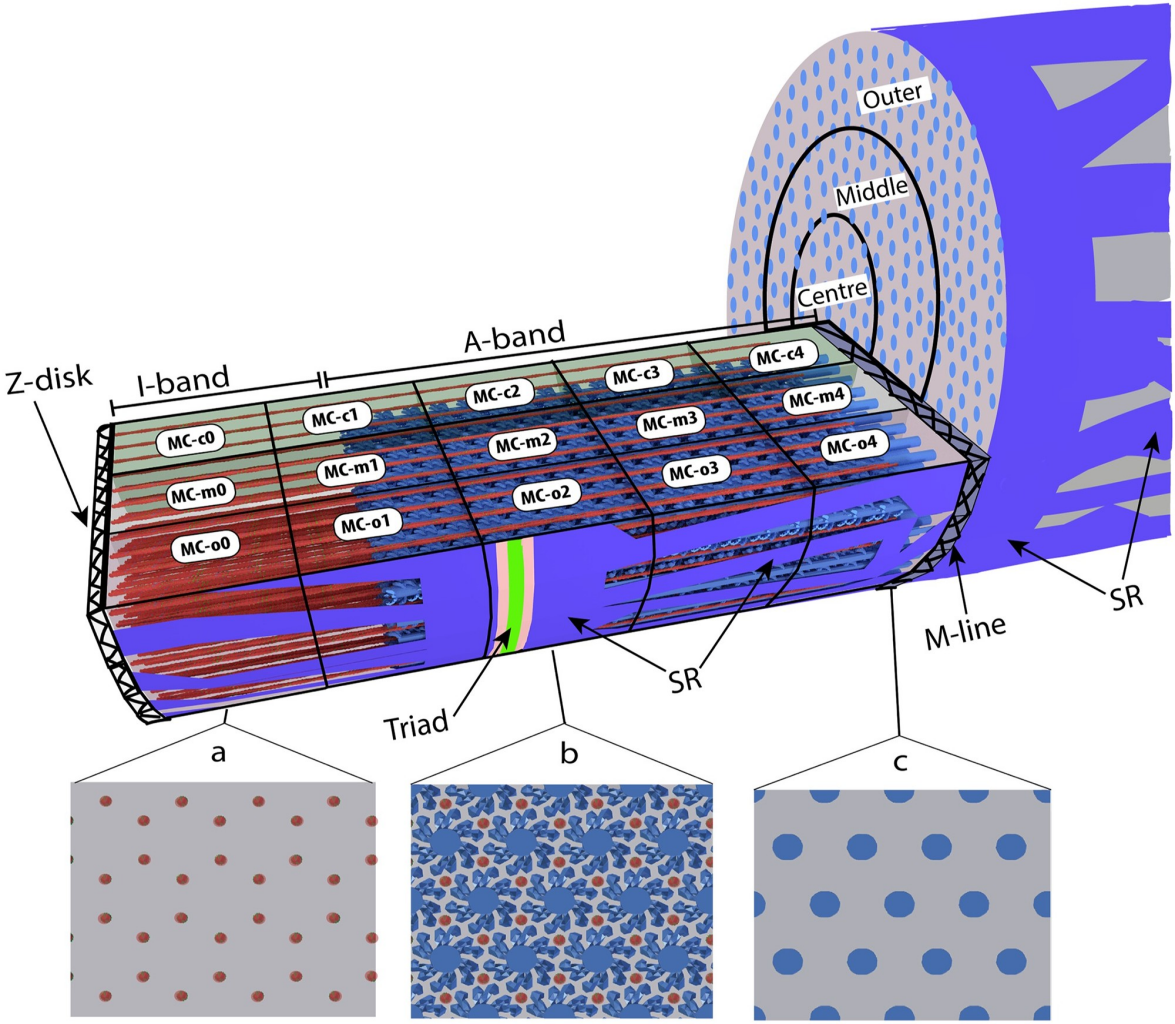
1/8th wedge 3D-model of a half sarcomere. Wedge-shaped sarcomere model with filaments at optimal overlap 2.3 μm length, showing location of Triad, SR, Z-disk, I-band, M-line, and A-band. Panels: a, b, and c, show how the sarcomere would appear in cross section at the three locations, illustrating how myofilament packing varies along the sarcomere: a) slice containing only actin filaments (I-band), b) slice of overlapped myosin and actin filaments (A-band), showing the myosin heads projecting off the filament backbone in pairs and c) slice near middle (M-line) of sarcomere in myosin head bare zone. Thick myosin filaments illustrated in blue, thin actin filaments illustrated in red. Virtual measurement compartments (MC-c0, MC-m0… etc) created to count changes in ion distribution, labelled on surface of compartment where they occur.

Within each sarcomere, the actin and myosin filaments are arranged in a myofilament lattice (MFL). Myosin filaments are in the middle of the sarcomere and project towards the Z-disks which are formed by intermediate filaments and provide the transition to the neighbouring sarcomere. The actin filaments are anchored in the Z-disk and project towards the middle of the sarcomere (Fig 1). When the sarcomere contracts, the Z-disks at either end are pulled toward the centre of the sarcomere. Within mammalian skeletal muscle the MFL is arranged most commonly so that actin and myosin filaments are inter-spaced in a complex repeating hexagonal pattern so that each myosin filament is surrounded by 6 actin filaments, each of which is neighboured by 3 myosin filaments (Fig 1, S1 and S2 Figs). It is within this highly-organized structure, or micro-architecture, that Ca^2+^ ions need to diffuse and interact with the various proteins, buffers, and pumps, including the proteins that regulate muscle contraction. Additional proteins, titin and nebulin are also present in the sarcomere, but these are not directly represented in our structural model.

The SR is a fenestrated (windowed) sac-like membrane, which surrounds the myofibril and stores large amounts of Ca^2+^. In mammalian skeletal muscle, the SR is intersected by the T-tubules roughly 500 nm from the z-disk and forms a complex referred to as the triad. The area of the SR which contacts the t-tubule is referred to as the terminal cisternae. During activation, Ca^2+^ ions are released from the terminal cisternae through the ryanodine receptors (RYR) to diffuse through-out the myoplasm. At the same time ATP powered Ca^2+^ pumps (SERCA) embedded in the SR continuously remove Ca^2+^ from the myoplasm, pumping Ca^2+^ back into the SR where it can eventually be released again. As the SR accumulates Ca^2+^, myoplasmic calcium concentration ([Ca^2+^]) falls.

### Limitations of experimental measurement of changes in calcium concentration

The rise and fall of free [Ca^2+^] within individual muscle cells, or the calcium transient, is measured experimentally using a Ca^2+^ sensitive fluorescent indicator such as furaptra or mag-fluo-4 [1]. The measured fluorescence is then used to estimate changes in [Ca^2+^] using a spatial averaging technique [1–4]. While this has been an extraordinarily useful tool for studying Ca^2+^ transients in muscle fibres, this method has limited spatial and temporal resolution [1–3, 5].

To address these limitations and to gain further understanding of how diffusion and binding of Ca^2+^ occur within an individual sarcomere, researchers have developed computer simulation models that attempt to overcome the spatial and temporal limitations of direct measurement. Cannell and Allen [5] developed the first 3D multi-compartment sarcomere model to examine diffusion of Ca^2+^ in frog muscle. The multi-compartment half sarcomere simulation has been further developed, and refined over time by Baylor and Hollingworth [2, 3, 6]. In general, these Ca^2+^ diffusion simulations, divide the sarcomere into a 3-dimensional array of smaller compartments, and use a series of ordinary differential equations (ODE) to represent the diffusion, binding, and pumping reactions that occur within and between the compartments. Together, these sarcomere simulations have proven to be valuable tools for the identification of the large localized concentration gradients that exist during muscle activation, the importance of Ca^2+^ buffers such as ATP, and for identifying experimental errors that can arise when using calcium indicators [1–6].

As with any technique or tool, there are inherent limitations with using ODEs to power a simulation, as described by Kerr [7] and Franks [8]. While ODE simulations can be computationally efficient, simulations based on ODE’s are deterministic and describe the mass action of the system. These limitations can be problematic when used in biological systems which are intrinsically stochastic, and the volumes and ionic concentrations are small [7]. An example of an ODE calculation error can be demonstrated in a half sarcomere model where the resting concentration of Ca^2+^ is 50 nM [1–6]. This resting [Ca^2+^] translates to 22 Ca^2+^ ions within the half sarcomere model. If that half sarcomere is then virtually divided into 4 compartments along each of the x, y, and z axes, the ODE simulation will calculate the concentration in each compartment as 50 nM which translates to about 0.34 ions per compartment. Whereas in reality, some compartments would contain one or possibly two Ca^2+^ ions, while others would be empty. More importantly though, models based on ordinary differential equations do not simulate the diffusion of ions, but simply calculate the transfer rates between compartments and reactions that occur within each compartment. Due to this limitation, it is very difficult to develop this type of simulation to be sensitive to the structural micro-architecture within the sarcomere, let alone the differences in filament distribution within that micro-architecture.

As previously stated, skeletal muscle has an intricate and complex structure. The myofilament lattice is highly organized with several proteins organized into filaments which have a specific arrangement and relationship to each other within the sarcomere (Fig 1) [9–11]. Additionally, the SR surrounding the myofilament is irregularly patterned [12, 13]. While stochastic simulations of myosin and actin interaction have been developed [14], and it has been demonstrated mathematically that the myofilament lattice produces an obstruction to the diffusion of Ca^2+^ ions [15], to date a simulation of Ca^2+^ diffusion within the myofibril that incorporates the topological representation of the myofilament lattice or micro-architecture of the sarcomere has not been developed.

It is the purpose of this paper to create a structural model of a skeletal muscle sarcomere and develop a simulation to explore the potential impact the micro-architecture of the sarcomere has on the diffusion, binding and uptake of Ca^2+^ ions during a single activation. This simulation will also produce visualizations of this process. Due to the topological structural complexity within the sarcomere, the sarcomere model will be limited to a 1/8th wedge of the half sarcomere. The model produced is static and does not incorporate any filament compliance [16] or cross bridge binding. The model will further be divided into 15 measurement compartments labeled MC-c0—MC-o4 (Fig 1) which will allow us to count ions and reactions that occur within these compartments. We will accomplish this by first building a structural representation of the sarcomere using the software tool Cellblender, then incorporate the model into MCell software (Monte Carlo Cell), as developed and described by Stiles [17] and Czech [18]. We will then run a series of stochastic simulations using MCell to simulate the release and diffusion of Ca^2+^ and the reactions between Ca^2+^ and the fixed and diffusing Ca^2+^ buffers (Troponin-C and ATP) within the sarcomere during a simulated single activation. A complete and detailed description of the model and its construction is described in the Methodology section.

## Results

We were able to succesfully build our 1/8th half sarcomere model, and run 10 seeded simulation runs. The completed model contained 120 actin filaments, 47 full myosin filaments, and 24 half-myosin filaments. The centre-to-centre distance between the myosin and actin filaments (where they were overlapped) in the model was 25.9 nm (Eq 1). Ca^2+^ was released into the simulation via a simulated action potential and diffused at a rate of 3x10^-6^ cm^2^s^-1^. ATP concentration was simulated at 8 mM and diffused in the model at rate of 1.4x10^-6^ cm^2^s^-1^ [6]. As only a 1/8th wedge of the sarcomere was modelled, the boundary of the slice was governed by a standard MCell reflection rule. This rule is based on the assumption that when any model element diffuses from the model another will enter. The diffusion rate coupled with the simulation time step resulted in an average diffusion step size for Ca^2+^ of 0.6 nm, and 0.18 nm for ATP, insuring reactions were not missed.

### Measurement compartments

We subdivided the interior of the model into 15 measurement compartments (MCs). These MCs were transparent to the molecules and ions within the simulation, but enabled the measurement of molecules, ions, and reactions that occurred within their boundaries. Unlike the previous simulations of Cannell and Allen [5] and Baylor and Hollingworth [2, 3, 6], in which all the compartments maintained the same volume, we divided our sarcomere model evenly so that each MC was 1/5th the length and 1/3rd the radius of the sarcomere model (Fig 1). The resulting concentric radial slices were referred to as: center (MC-c), middle (MC-m), and outer (MC-o). The compartments were then numbered sequentially from the Z-disk, beginning with 0 and ending with 4 closest to the M-line. In contrast to previous simulations, where concentration of Ca^2+^ was assumed to be uniform throughout a compartment, our model allowed a continuous concentration gradient across any MC. Diffusion was simulated independent of the boundaries of the MCs. The number of ions within the measurement compartments at each time step and the fluid volume of the respective compartment were used to calculate concentration.

### Compartment fluid volumes

The placement of the actin and myosin filaments within the sarcomere model displaced fluid from the measurement compartments (MCs); the corrected fluid volume of the MCs is presented in Table 1. The completed model contained a total of 1807742 mesh faces. Simulation runs took on average 15 days to complete. Each simulation run of our model used an average of 17.6 E+10 random numbers, and conducted an average of 18.8 E+12 ray-polygon intersection tests, which result in an average of 21.8E+8 ray-polygon intersections. These numbers relate not only to the complexity of the simulation, but convey the number of interactions that occurred between the various elements within the simulation.

$$\begin{array}{c} d_{10}=\sqrt{\frac{LV}{\frac{2}{\sqrt{3}}\times SL}} \end{array}$$ (1)

Where:

d_10_ = the standard measure of lattice spacing from x-ray diffraction,

LV = the lattice volume, and

SL = sarcomere length.

**Table 1:** Fluid volume of measurement compartments.

Compartment:	MC-0	MC-1	MC-2	MC-3	MC-4
MC-c	2.35E-03	2.07E-03	1.77E-03	1.77E-03	1.79E-03
MC-m	7.11E-03	6.39E-03	5.61E-03	5.61E-03	5.67E-03
MC-o	1.22E-02	1.08E-02	9.37E-03	9.37E-03	9.46E-03

Measured in μm^3^, calculated as: volume of compartments minus the volume of the protein filaments within the compartments at sarcomere length 2.3 μm

### Simulated action potential

**Fig 2:**
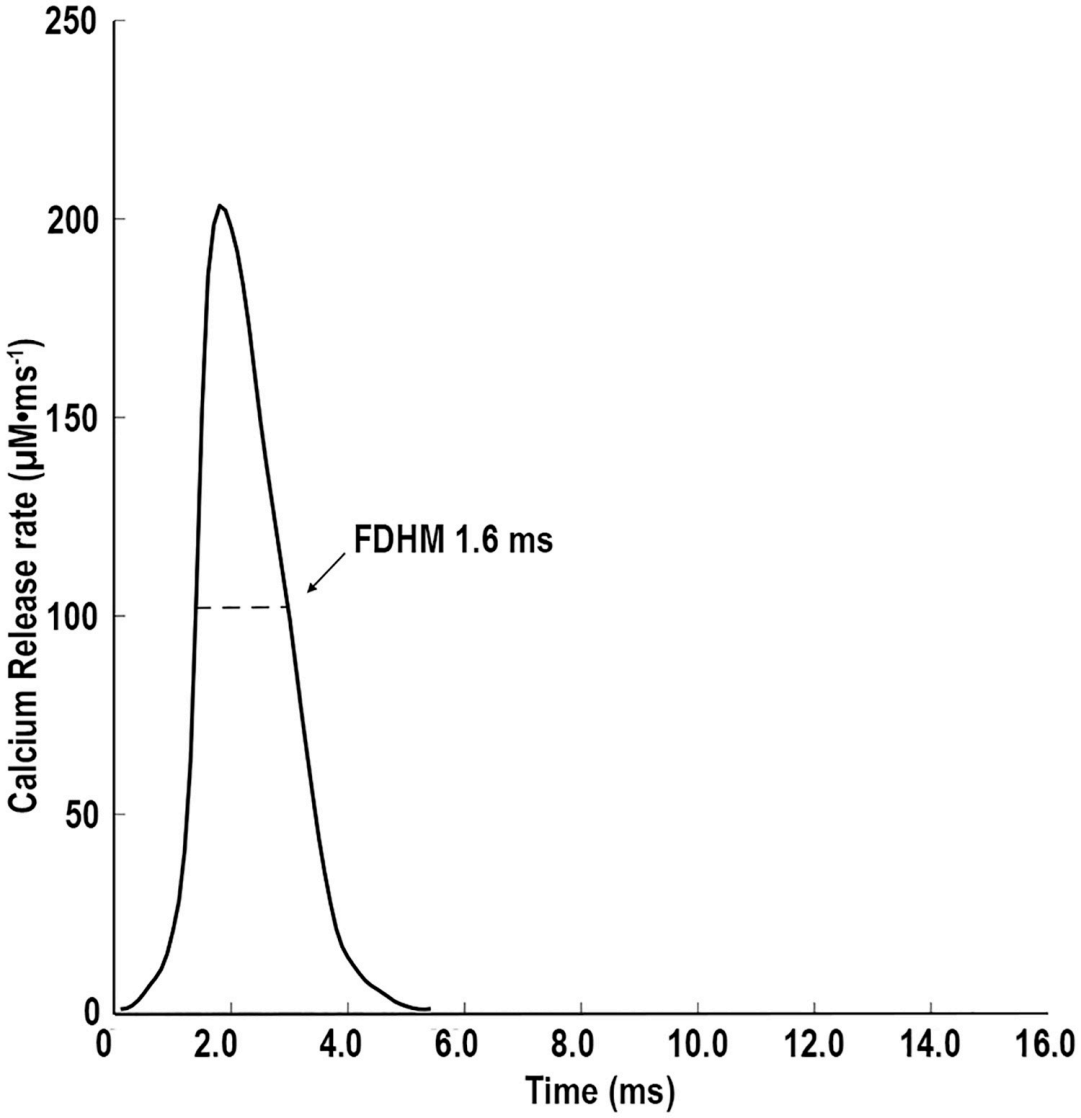
Rate of Ca^2+^ release. Release rates used to drive Ca^2+^ release, through the RYR in the model. The rate was changed every 1/10^th^ of a ms during the simulation.

The release of calcium ions through the RYRs was driven by a changing reaction rate derived from the action potential equation from Baylor and Hollingworth [6]. Peak release rate of Ca^2+^ into the sarcoplasm was 205 M ms^-1^ and the full duration at half maximum (FDHM) was 1.6 ms (Fig 2). The total number of Ca^2+^ ions released, represented a [Ca^2+^] that would equal 343.3 μM within the whole model if no binding or uptake occurred.

### Changes in calcium concentration

**Fig 3:**
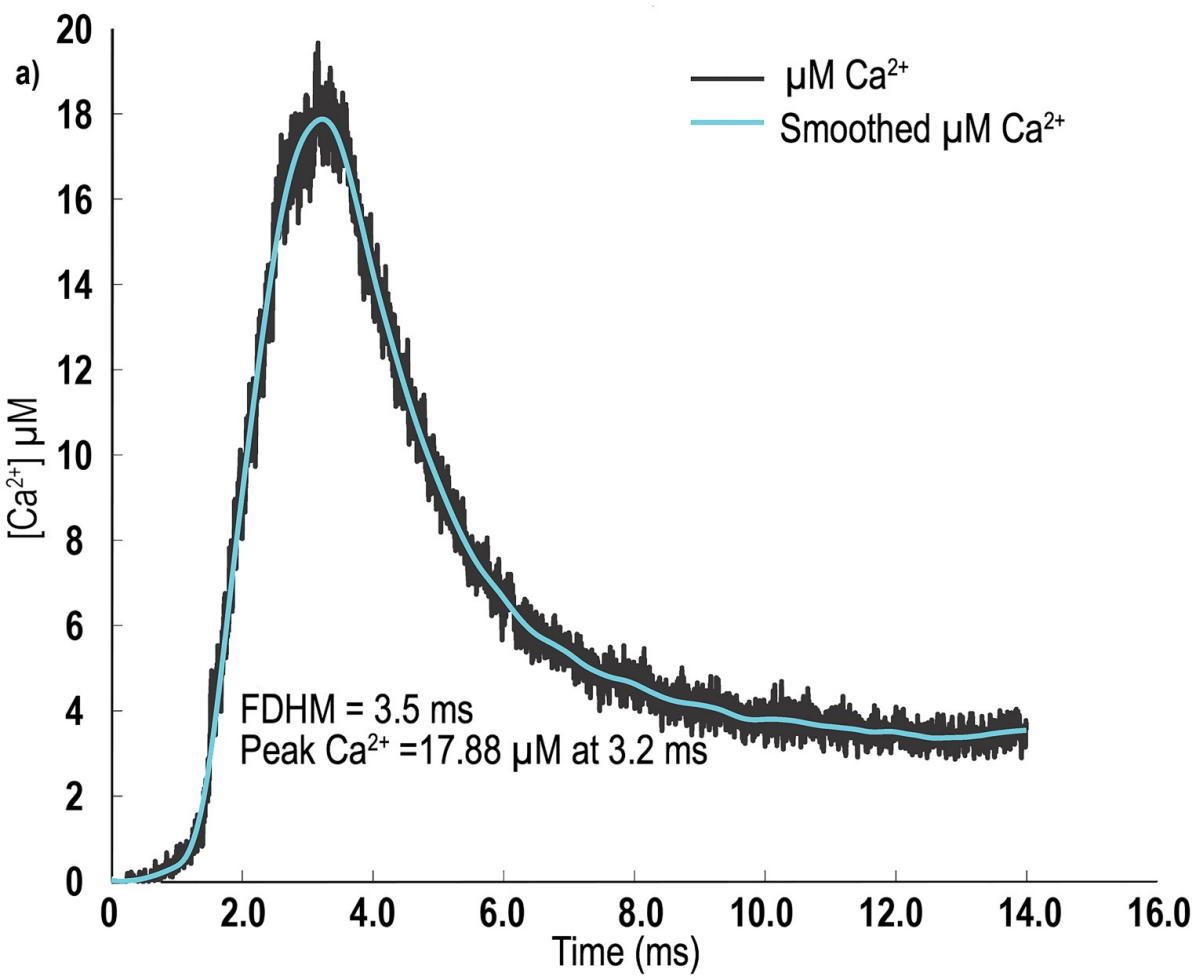
[Ca^2+^] transient plots from our model. The figure is a data plot for a single seed value (seed 10) used in the experiment plotted with line of best fit using a robust local regression using a weighted linear least squares (rloess) smoothing function.

In our simulations, we observed an average peak [Ca^2+^] of 17.43 ± 0.87 μM. The average FDHM of the change in [Ca^2+^] was 3.36 ± 0.16 ms. In Fig 3, we plot the change in [Ca^2+^] within the whole sarcomere model over time, for seed value 10. These values are within the ranges reported by Baylor and Hollingworth (Δ [Ca^2+^] 16.1 μm for their model, and 17.5 μm for furaptra determined Δ [Ca^2+^]) [6] for both experimental results, from mouse fast twitch EDL fibres at 16° Celsius, and their multi-compartment sarcomere simulation.

In Table 2. we present the average and standard deviations, for all 10 simulation runs with respect to peak [Ca^2+^], and the time at which [Ca^2+^] peaked. It should be noted that, as our simulation was based on a structural model of the sarcomere, the fluid volume differed in the various MCs (Table 1). While we made sure that each of the MCs within longitudinal sections were of equal size, the positioning of the actin and myosin filaments within the model meant that MCs at either end had different fluid volumes. Myosin filaments originate in MC-4, continue through MC-2, but extend only partially into MC-1, and do not occupy MC-0 at all (Fig 1), whereas actin filaments that originate in MC-0, continue through to MC-3 and only extend partially into MC-4 (Fig 1). The end result is that the fluid volume is only the same in MC-2 and MC-3, which also have the smallest fluid volume. MC-0 has the greatest fluid volume, followed by MC-1 and MC-4, respectively (Table 1).

**Table 2:** Peak [Ca^2+^] within the individual MCs in the model.

	MC-0	MC-1	MC-2	MC-3	MC-4
MC-c					
[Ca^2+^] μM	12.75±0.94	16.37±1.57	19.15±1.39	12.83±0.79	8.72±0.76
# Ca^2+^	18 ± 1	20 ± 2	20 ± 1	14 ± 1	9 ± 1
Time (ms)	3.99±0.30	3.67±0.30	3.56±0.30	3.56±0.24	4.23±0.23
MC-m					
[Ca^2+^] μM	13.23±1.10	19.71±0.92	25.69±1.28	14.13±0.76	7.79±0.61
# Ca^2+^	57 ± 5	76 ± 4	87 ± 4	48 ± 3	27 ± 2
Time (ms)	3.76±0.23	3.28±0.16	3.15±0.16	3.36±0.23	3.96±0.30
MC-o					
[Ca^2+^] μM	13.08±0.84	29.81±1.42	54.55±2.41	16.72±1.12	6.75±3.81
# Ca^2+^	96 ± 6	195 ± 9	308 ± 94	94 ± 6	39 ± 3
Time (ms)	3.57±0.20	2.88±0.15	2.54±0.17	3.28±0.20	3.81±0.28

Peak [Ca^2+^], average number of ions present within each compartment and time peak was reached in each compartment (mean value ± standard deviation) n = 10.

**Fig 4:**
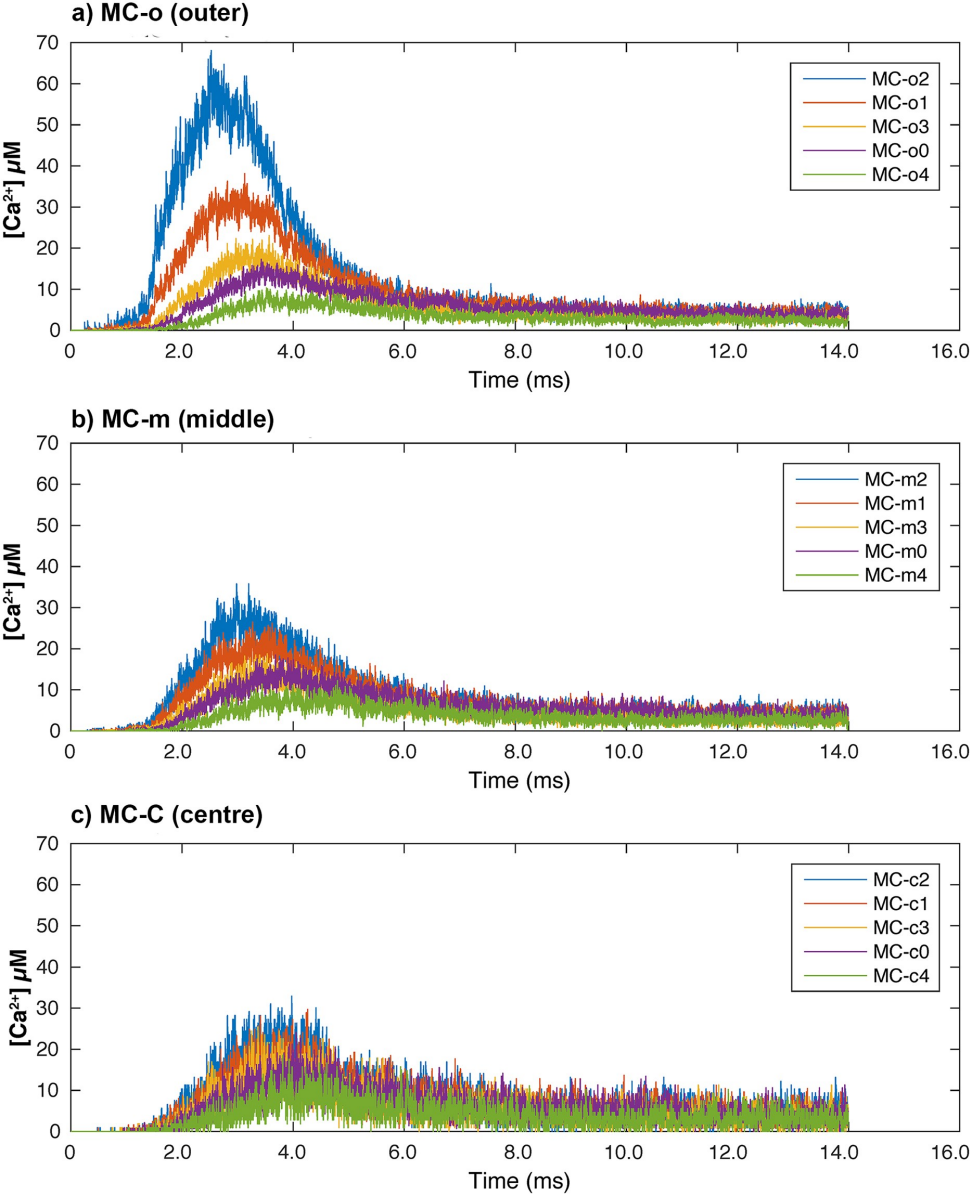
Calcium concentration along the sarcomere. (a) Presents the 5 plots from MCs in the outer radial shell of the sarcomere model. (b) Presents the 5 plots from MCs in the middle radial shell of the sarcomere model. (c) Presents the 5 plots from MCs in the centre radial shell of the sarcomere model. Within the legend all results are sorted according to distance from the RYR’s to centre of each compartment.

For a single simulation run (seed 10), we plotted the [Ca^2+^] over time within each of the individual MCs (Fig 4). In Fig 4a, the [Ca^2+^] in the five outer MCs (the MCs that are bordered by the SR and Triad) are depicted. In Fig 4b, [Ca^2+^] within the middle five MCs of the sarcomere are plotted, and finally in Fig 4c, we plot [Ca^2+^] within the five centre MCs of the sarcomere model.

When we examine the plots in Fig 4 along with Table 2, we see that the general trend is that peak [Ca^2+^] within the MCs decreases and occurs later, as their distance from the release site increases. In MC-o2, which lies under the triad, [Ca^2+^] peaks at 54.55 μM, whereas in MC-c4 which is the furthest away from the triad and positioned closest to the middle of the sarcomere, the [Ca^2+^] reaches only 8.72 μM. Further examination of Table 2, and Fig 4, shows a slight bias, where more Ca^2+^ ions have diffused towards the Z-disks and less towards the M-line.

### Calcium bound to troponin-C

In our simulation, we saw concentration of troponin-C (TnC) bound with Ca^2+^ (single and double bound states were grouped together) within the whole model peak at 230 μM, with a standard deviation (SD) of 1.5 μM. These results are again very similar to the simulation results of Baylor and Hollingworth [6].

Within the individual MCs in the simulation, we are presenting our data in terms of percentage of available TnC binding sites for Ca^2+^, grouping single- and double-bound states together. We have done this as the number of TnC binding sites varies between MCs, depending on where the MC intersects the actin filaments. As a result, the concentration of available TnC sites varies slightly between MCs. The MC with the lowest number of TnC binding sites is MC-4 (directly adjacent to the M-line) as only 2/3^rd^ the length of the MC is occupied by actin filaments. Presenting the data in terms of percentage of available binding sites allows a more direct comparison between the MCs within the model, and is more functionally relevant.

**Fig 5:**
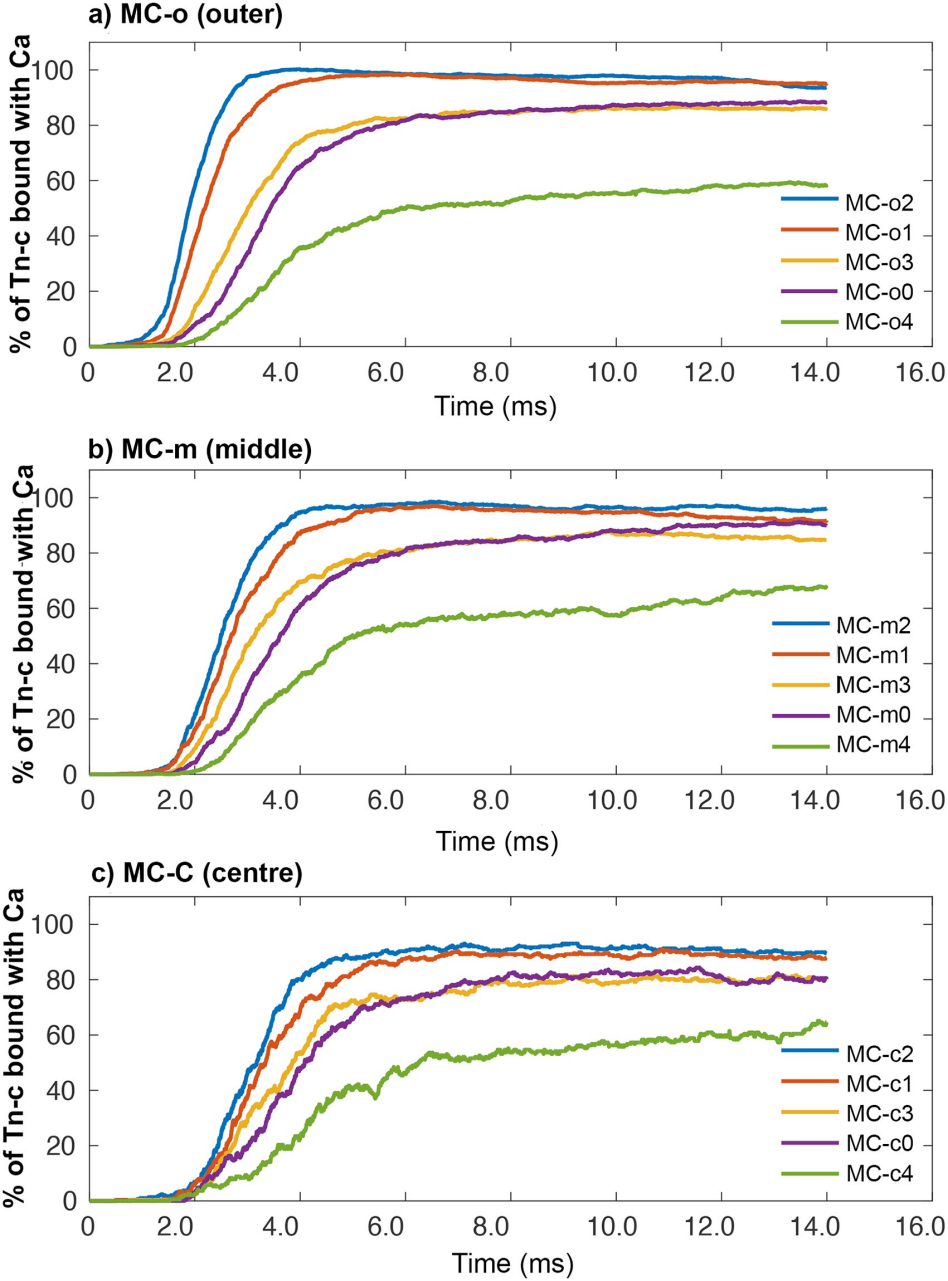
Percentage of available TnC binding sites bound with Ca^2+^. (a) plots from 5 MCs in outer radial compartments. (b) plots from 5 MCs in middle radial compartments. (c) plots from 5 MCs in centre radial compartments. Within the legend all results are sorted according to distance from the RYR’s to centre of each compartment.

In Fig 5, we present the percentage of TnC binding sites occupied with one or two Ca^2+^ ions over time for each of the MCs for seed 10 data. In MC-1 and MC-2 regardless of their radial position (o, m, c) ≥ 90% of the available TnC binding sites are occupied by Ca^2+^ (Fig 5a). In MC-0 and MC-3 the percentage changes to between 80% and 90% (Fig 5b), whereas in MC-4 (closest to M-line) the percentage of occupied TnC binding sites reaches just 60% regardless of radial position (Fig 5a, 5b and 5c).

### Visualizations of the simulation

**Fig 6:**
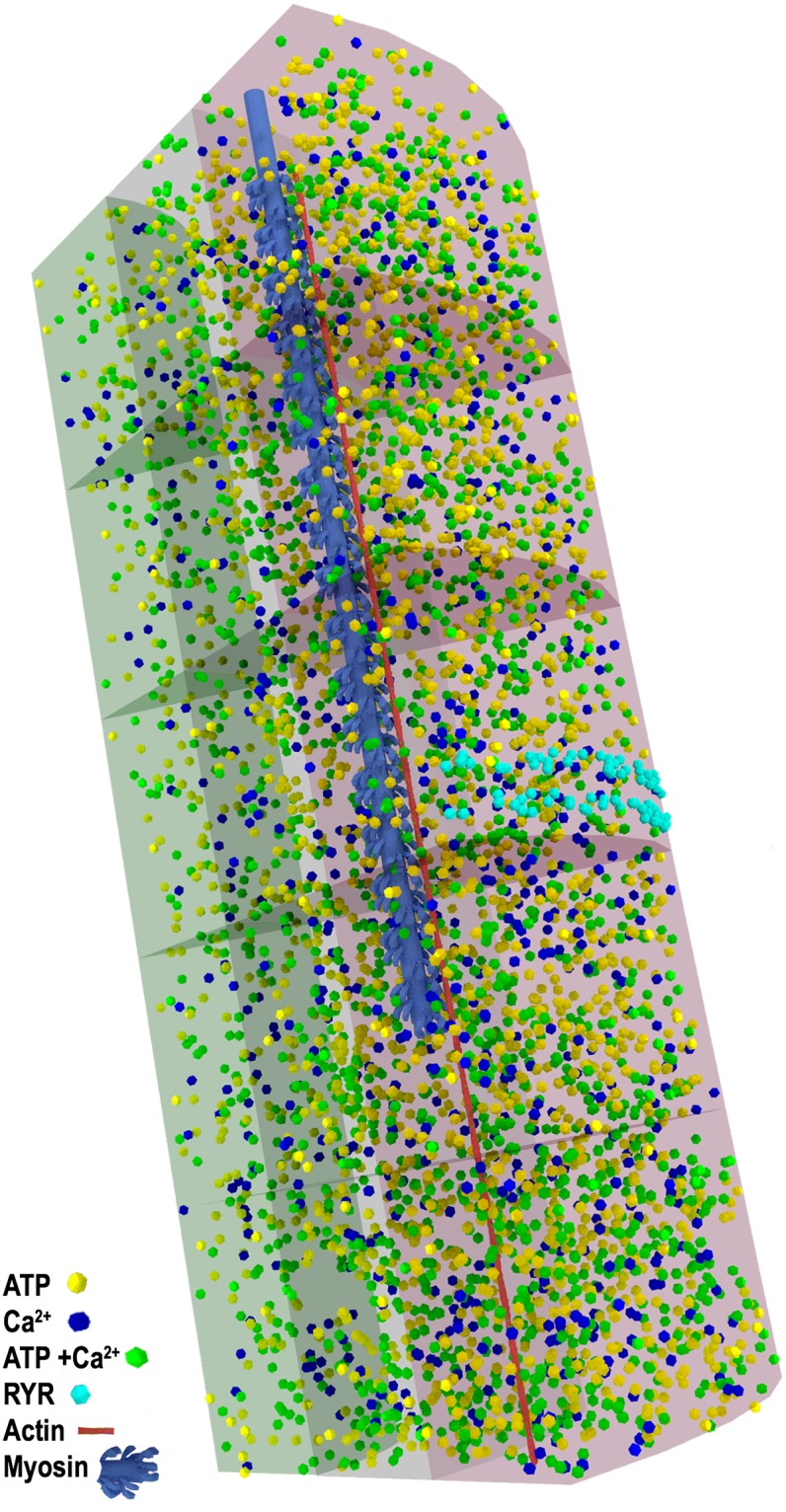
Model at peak [Ca^2+^]. Free Ca^2+^ ions are blue, ATP is yellow, ATP+Ca is green, RYRs are cyan. A single myosin filament (blue) and actin filament (red) are rendered for scale and position. Ions are depicted much larger than scale for visibility, MC-o translucent red, MC-m translucent grey, MC-c translucent green. Same renderings as S1 Video.

**Fig 7:**
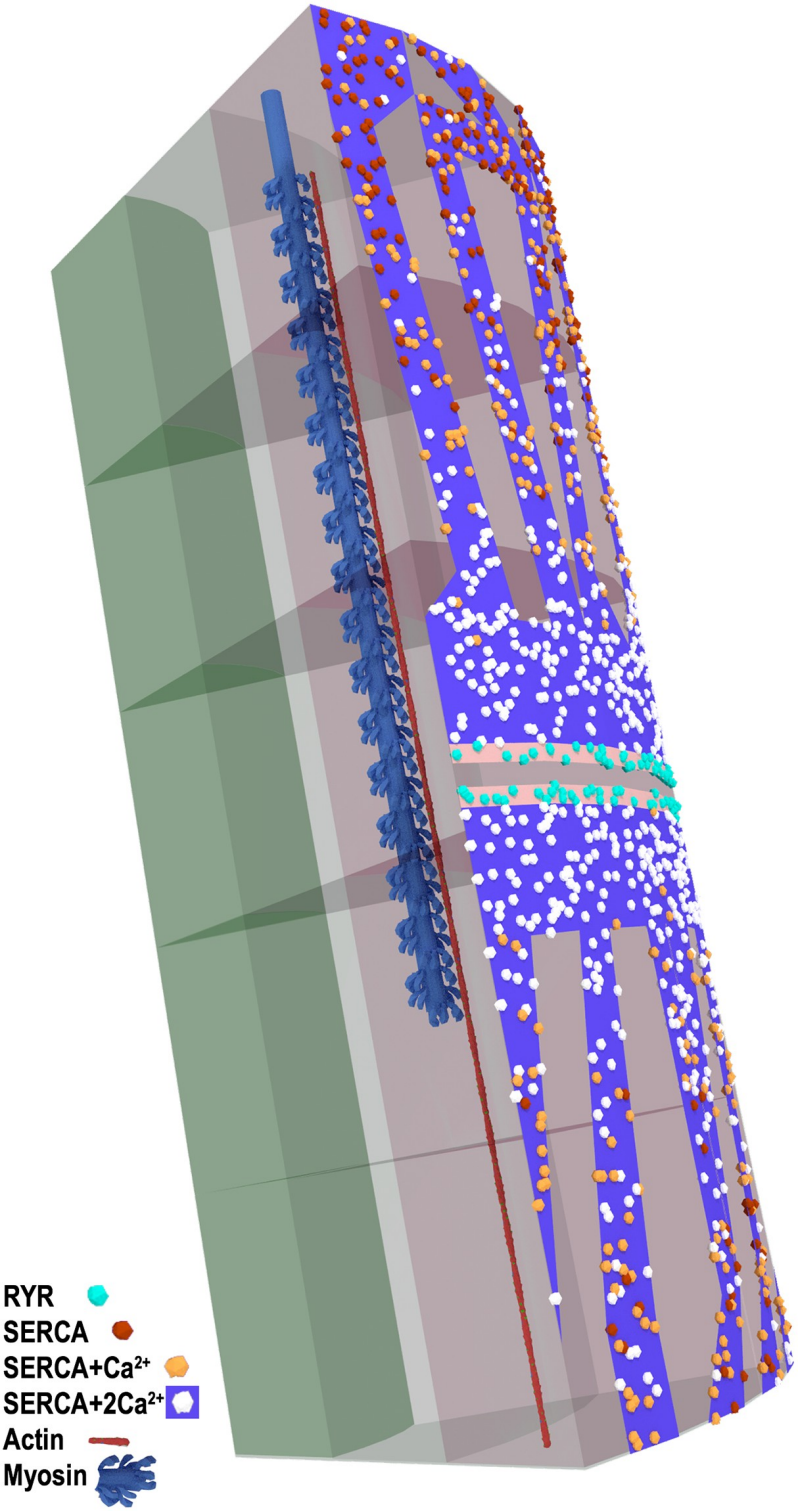
Fenestrated SR, SERCA activation at peak [Ca^2+^]. SERCA pumps bound with 2 Ca^2+^ ions are white, SERCA bound with a single Ca^2+^ are orange, unbound SERCA are dark orange, RYR release sites are cyan (single myosin and actin rendered for scale).

**Fig 8:**
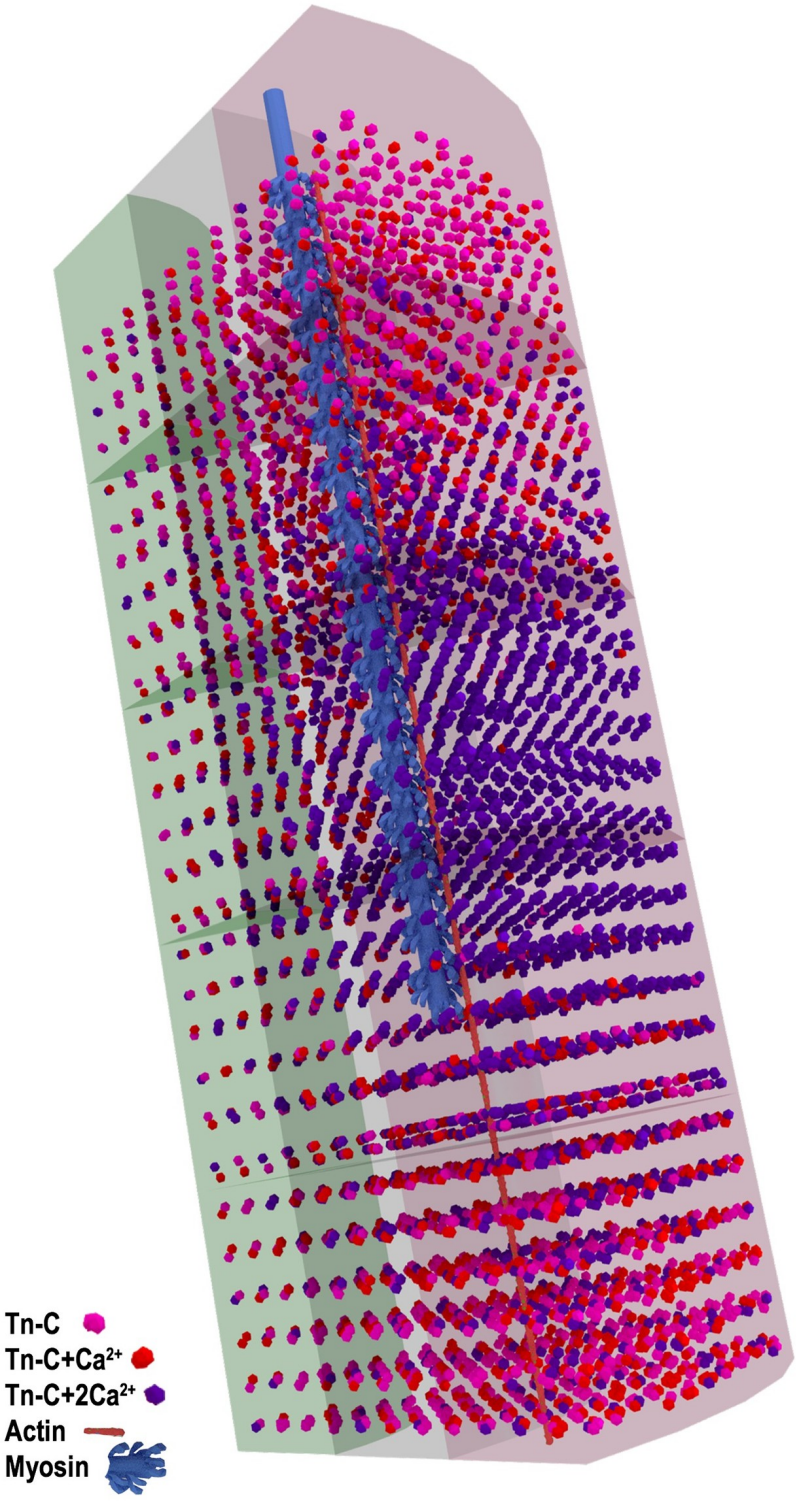
Model at peak [Ca^2+^] showing troponin-C binding sites. TnC bound with 2 Ca^2+^ ions dark purple, TnC bound with single Ca^2+^ ion red, TnC unbound pink (Same renderings as used in S2 Video).

A unique benefit of computer simulation is that we are able to visualize our model at any time, and examine in great detail the position and binding state of all the Ca^2+^ ions: free, bound to ATP, or TnC, within the model. In Figs 6, 7 and 8, we present still images of the simulation at peak [Ca^2+^] which occurs at 3.2 ms. We render the simulation to highlight free Ca^2+^, ATP, and ATP+Ca (Fig 6), the binding state of the SERCA pumps (Fig 7), and the binding state of TnC (Fig 8). Single myosin and actin filaments are rendered within the model to assist with orientation of the sarcomere.

In Fig 6, it is easy to see how Ca^2+^ ions are still clustered in the MCs closest to the release site on the triad. This image also illustrates the very large amount of ATP that is present throughout the sarcomere and reveals the very large amount of Ca^2+^ that is buffered by ATP at the time of peak [Ca^2+^]. In the supplemental information we present a complete animation of the seed 9 simulation run S1 Video. In this animation, diffusion of Ca^2+^, ATP, and ATP+Ca are presented with run time for the simulation.

In Fig 7, we show the SR and the SERCA pumps in the model at 3.2 ms, and it can be seen at this time that the pumps that are the closest to the triad release site are all completely saturated, with each SERCA pump bound with 2 Ca^2+^ ions. Moving towards either the Z-Disk or the M-line, we start to see SERCA pumps in these locations still in the unbound or single-bound state.

In Fig 8, we show the model highlighting TnC bound with either one or two Ca^2+^ ions at the time of peak [Ca^2+^], 3.2 ms. At this time, free [Ca^2+^] is decreasing as the Ca^2+^ ions continue to be bound to buffers including available binding sites on TnC. For this reason, TnC+Ca will peak nearly 2 ms after peak free [Ca^2+^] at 5.2ms (Fig 5). It is easy to understand that at this time many of the available TnC binding locations have been bound with Ca^2+^ ions and that the transition between single and double bound states is very short due to the high binding constant between TnC and Ca^2+^ ions. In supplemental information S2 Video we present a complete animation of the simulation, highlighting Ca^2+^ diffusion and binding to TnC locations on the actin filaments.

## Discussion

In this study, we have developed a novel multi-compartment model of the skeletal muscle sarcomere that incorporates the 3-dimensional structure of the myofilaments. Using this model and the simulation software MCell, we were able to run a series of stochastic simulations for the movement and binding of Ca^2+^ ions within the sarcomere following an action potential. Within this simulation, we were able to track the diffusion, binding, and uptake of Ca^2+^ ions, during a single muscle activation. Reaction rates and diffusion constants for our simulation were from published results of Baylor and Hollingworth [6]. The structural components of the model were based on the anatomical descriptions of the sarcomere from published data [10, 13, 19–21]. Models for protein filaments actin and myosin within the model were developed from models in the protein databank, and high resolution imaging studies [22–26].

Our structural model and simulation differ from previous simulations in that ours is stochastic, and it incorporates features of the MFL into the simulation. In addition to being able to evaluate the effect the MFL has on the diffusion of Ca^2+^ ions, this type of simulation also produces visualizations of the calcium transient during a single activation process. This feature may lead to greater insights and understanding of excitation-contraction coupling. We should note, as a testament to the robustness of the model of Baylor and Hollingworth [6], we did not alter or adjust the reaction rates that they determined but used them within our model to achieve similar results. However, our results also allowed us to evaluate the impact of myofilament structure on the diffusion of Ca^2+^.

It is important to recognize that our model has numerous limitations: the model is currently only a 1/8^th^ wedge of the sarcomere, and we used a reflection rule at the model boundaries, reflecting diffusing ions and molecules, with the assumption that roughly the same number of ions would exit as enter the model. The modelled proteins and filaments are also static, and we did not model the compliance of the myofilaments, or the movement of the myosin S1 heads. While we were unable to included these aspects within our model at this time, we realize that these elements will further affect MFL density and therefore diffusion in the model.

In this study we primarily focused on demonstrating the stochastic model’s ability to repeat previous experimental results. However, the asymmetrical binding of Ca^2+^ to TnC along the actin filament that was demonstrated in our study provides a mechanism to further explain why single stimulation approximating a twitch, would result in sub-maximal force production [27]. This observation also provides further mechanistic support to the hypothesis that shortening induced force depression, is not a result of reduced actin and myosin binding opportunities, as more actin binding locations will be accessible towards the Z-disk as the filaments continue to overlap, but more likely related to geometric changes within the MFL [28].

It is our hope that the further development of this type of stochastic model, will provide a means to explore how genetic disorders, which affect the structure of protein elements within the the skeletal muscle sarcomere, contribute to changes in muscular function and result in myofibrilar myopathies. With this type of sarcomere model it should be possible to accomplish this by modelling the physical structures present in the disease condition, and then noting their affect on Ca^2+^ diffusion and distribution.

### Comparing our results with experimental and simulation data

When we compare the results we obtained for the continuous diffusion of Ca^2+^ through the sarcomere to both the spatially averaged results from experimentation and simulation results of Baylor and Hollingworth [6], we see very good agreement between the three data sets. The results from our simulation (Fig 3) closely follow both the experimental and simulation results for the change in [Ca^2+^] over time as obtained by Baylor and Hollingworth [6].

As our simulation model was stochastic, we ran our simulation ten times with different random number seed values, this process gave us a data set from which we were able to run some simple descriptive statistics. From the 10 simulation runs, our model returned an average peak [Ca^2+^] of 17.43 μM with a SD of 0.86 μM, the plot of the [Ca^2+^] had a FDHM of 3.36 ± 0.16 ms (Fig 3). These values are very close to the peak [Ca^2+^] values of 17 μM with a FDHM of 3.6 ms, as reported by Baylor and Hollingworth [6] for their simulation (at length 2.4 μm), as well as their direct experimental results for change in furaptra-detected [Ca^2+^] of 17.5 μM and FDHM 4.5 ms (at a length of 3.5 μm). Not only did our simulation replicate the general results, our graph of the change in average [Ca^2+^] over time (Fig 3) matches the graphs of Baylor and Hollingworth [6] for shape and magnitude. While the results from our simulation produced a slightly larger peak [Ca^2+^] and slightly narrower FDHM than the simulations of Baylor and Hollingworth [6], their results fell within the SD of our simulation results. One of the more interesting outputs from our stochastic model, was the model’s ability to generate an estimate of the SD that can be expected experimentally, this is not produced in previous ODE based models [5, 6], as they are deterministic. As our model is stochastic, the estimate of the SD is based on the variability of diffusion and the probability of reactions occurring within the simulation: this is a first for this type of modelling, and the variation is independent of variability in measurement.

### Changes in calcium concentration within the compartments

The greatest benefit of multi-compartment models is their ability to predict how Ca^2+^ ions will diffuse through the sarcomere and interact with various buffers and receptors at much greater resolution than is typically available in experimentation. While we were pleased that our simulation model was able to replicate the experimental and simulation results for the general [Ca^2+^] transients [6], we expected the distribution of Ca^2+^ between the compartments of our model to differ from previous models. There are two related reasons why we expected our simulation to produce slightly different results. The first of these is that our simulation contains the myofilament structures, and we expected that the inclusion of the myofilaments would influence the diffusion pattern of the Ca^2+^. As our model simulates the diffusion of Ca^2+^ directly, the pattern that the diffusing ions ultimately follow is influenced by collisions, interactions, and reactions within the simulation. As such, our simulation is continuous without regard for compartments, unlike previous attempts to model Ca^2+^ in a sarcomere. The second reason we expected our results to be different is that the compartments we used to determine how Ca^2+^ is diffusing and reacting within our simulation model are of equal dimensions, and not of equal fluid volume. In the mathematical models used by Baylor and Hollingworth [6] and others [2, 3, 5], all compartment volumes within the simulations are equal. To create equal volumes between the concentric cylindrical compartments, the radial width of the compartments needs to decrease as you move from the central cylinder to the outer cylinder. This must occur to balance the increased circumference of the cylinder as you move outward. This results in a radially thin outer cylinder with a large circumference, and a radially thick central cylinder with a small circumference. While there are practical reasons for maintaining equal volumes between all measurement compartments in a mathematical simulation, it creates problems in a structural model, and is not necessary.

In a structural model, the asymmetrical distribution of the myofilaments will naturally create asymmetrical volumes within the model. To compensate for these asymmetrical volumes the model would then require complex asymmetrical compartments to equalize the volumes. For our simulation, we chose to create our compartments using equal dimensions for radial width and length, and allowed circumference to vary by position of the cylindrical MC, knowing that the volume within each MC would change anyway depending on the unique composition of myofilament proteins within that MC (Table 2). The lack of equal volumes does not affect the diffusion or reactions of elements within our simulation though, as diffusion and reactions are simulated directly, and are independent of the MCs. It is also important to point out that within our simulation the reactions between Ca^2+^ and SERCA occur at the surface of the SR, where the SERCA pumps are bound, and are independent of other reactions occurring within the outer MCs. So for these reasons, we cannot directly compare the results from within the compartments of our structural model with those of previous models [3, 5, 6].

In our simulation model, we were particularly interested in how the inclusion of the myofilaments into our sarcomere simulation would affect the diffusion and subsequent distribution of Ca^2+^ following an activation. Examining how the [Ca^2+^] differed between the MCs in our simulation, we see support for the assertions of Shorten and Snyed [15] that the structure of the myofilament lattice impedes the diffusion of Ca^2+^ ions. Looking at the data in Table 2, we see that the diffusion of Ca^2+^ in our simulation is biased towards the Z-disks, and is not uniform in both directions. This bias is most notable in the central and middle MCs when we compare the [Ca^2+^] in MC-0 and MC-3.

The triad in our model is positioned 500 nm from the Z-disk as reported by Brown [20] as well as Gomez [29]. As each MC is 230 nm in length, the triad in our model is positioned just above MC-o2. Due to this position of the triad over MC-o2, the changes in [Ca^2+^] after a simulated release are greatest in this compartment. However, when we compare the distances from the release site to the centres of the MC-0 and MC-3 compartments, we see that the centre of the MC-3 compartments are closer to the triad than the centres of MC-0 compartments. As MC-3 is closer to the release site, we would expect that the peak [Ca^2+^] should be higher in these compartments than in MC-0, and that peak [Ca^2+^] should occur sooner. We would expect this, because diffusion should distribute Ca^2+^ evenly in both directions based on distance and time. We see this result only in the outer most compartments where peak [Ca^2+^] is higher and occurs sooner in MC-o3 than in MC-o0. However, when we look at compartments in the middle and centre of the model, we see no difference between peak [Ca^2+^], between MC-m0 and MC-m3, or between MC-c0 and MC-c3. This occurs even though the time to peak [Ca^2+^] remains as one would expect, with peak [Ca^2+^] occurring sooner in the compartments MC-3 (MC-o3, m3, c3) than in MC-0 (MC-o0, m0, c0)(Table 2). The idea that diffusion is being affected by the myofilaments, is further supported by the realization that MC-0 also has a slightly larger fluid volume than MC-3 (Table 2), and as a result more Ca^2+^ ions are present in MC-0 than are present in MC-3, even though it is further from the triad (Table 2).

If we consider just the number of ions present in a compartment, instead of the concentration of those ions, the effect the myofilaments have on the distribution of ions throughout the model becomes clearer (Table 2). From the result presented in Table 2, it is obvious that the distribution of the number of Ca^2+^ has been shifted towards the compartments with the least number of myofilaments, and the largest fluid volume. There are 2 mechanisms at work here. i) the myofilaments impede diffusion and ii) due to the lower volume, the concentration rises faster with fewer ions. Both factors would slow subsequent diffusion. The concentration differences tell us that the filaments impede diffusion. This is most apparent when we compare the number of ions at peak [Ca^2+^] in the MCs that are closest to the Z-disk MC-o0, MC-m0 & MC-c0 with the corresponding compartments MC-o3, MC-m3 & MC-c3 which are situated more toward the M-line and contain more myosin filaments (Table 2). Comparing these compartments, we see in the outer compartments that MC-o0 and MC-o3 have roughly the same number of ions. However, in the middle and central sections of the model, both MC-m0 and MC-c0 have significantly more Ca^2+^ ions than MC-m3 and MC-c3 respectively.

When we used a paired t-test to compare MC-m0 with MC-m3, and then MC-c0 with MC-c3, we saw that they are significantly different with a p = 1.27e-05, and p = 1.12e-07, respectively (Table 2). As the MCs towards the Z-disk have greater fluid volume and fewer filament structures, this seems to support the assertions of Shorten and Snyed [15] that the myofilaments can impede the diffusion of Ca^2+^. The reason that this occurs in both the middle and centre MCs and not in the outer MCs would also suggest that this effect is more pronounced as distance through the MFL increases. While it could be argued that the process of diffusion will balance the [Ca^2+^] rather than the number of Ca^2+^ ions present, this balancing would occur at equilibrium which is never achieved in our simulation.

### Troponin-C binding calcium ions

In our simulation, we also measured the concentration of TnC bound with Ca^2+^ (single and double bound states were grouped together). Within the whole model, peak TnC bound with Ca^2+^ peaked at 230 μM ± 1.5 μM at 5.2 ms (90% bound). These results again are very similar to the simulation results reported by Baylor and Hollingworth [6].

When we examine our simulation with respect to TnC binding Ca^2+^ within the MCs, we again see results that indicate that the inclusion of the myofilaments into our models affects not only the distribution of Ca^2+^, but also the binding of Ca^2+^ to TnC. In the MC-2 compartments (MC-o2, MC-m2, & MC-c2) which lie directly below the release site, the percentage of TnC bound with Ca^2+^ reaches 98% in o2 and m2, and 90% in c2. This peak occurs between 3—5 ms, then slowly starts to decline (Fig 5). When we then compare these results to the percentage of bound TnC in the neighbouring compartments MC-0, MC-1 and MC-3, we see a larger difference between these compartments than would normally be expected. Following MC-2, the highest percentage of TnC binding occurs next in MC-1, followed by MC-0, which is closely followed by MC-3 (Fig 5).

Peak TnC binding of Ca^2+^ occurs in the simulation at 5.2 ms. At this time, there is no difference in the percentage of bound TnC in either MC-o2 or MC-o1, with both compartments at 98%. There is also no difference in the percentage of TnC bound with Ca^2+^ in either MC-o3 or MC-o0 at 80%. As in Fig 4, we see that MC-o3 and MC-o0 have very similar percentages of bound TnC (Fig 5). This occurs in spite of the fact that MC-o0 is positioned further away from the release site than MC-o3. The further the Ca^2+^ has to diffuse through the MFL, the more the diffusion rate is slowed by the MFL. This is most noticeable in the very large decrease of binding that we see in MC-4, the series of compartments directly adjacent to the M-line in Fig 7. In these compartments (MC-o4, MC-m4, & MC-c4), we see that the percentage of TnC sites bound with Ca^2+^ peaks at only 60%, although it appears to still be slowly increasing as our simulation ends (Fig 5). The effect that the MFL has on Ca^2+^ and subsequently the percentage of bound TnC, is most apparent when we consider that the triad is located at 500 nm from the Z-disk. This means that regardless of the position of the MCs in our model, the triad is positioned very close to the middle of actin filaments, which are 1055 nm long in our simulation. If diffusion of Ca^2+^ was not being affected by the inclusion of the MFL in our model, we would expect to see a mirror image of binding of Ca^2+^ to TnC, one side reflecting the other. However, the presence of the MFL in our simulation results in there being more Ca^2+^ bound to TnC towards the Z-disk (MC-0) than towards the M-line (MC-4). This large difference in the percentage of TnC bound with Ca^2+^ in MC-4 that we see, is consistent in all simulations runs with different seed values, although only data from seed 10 are presented in Fig 5. It is important to realize that binding of Ca^2+^ to TnC on thin filaments that do not have adjacent myosin filaments, as is the case in most of MC-0, is inconsequential with respect to contraction. The impact of this would be expected to be greater at long SL.

### Conclusion

With our structural simulation model of a 1/8th half sarcomere, we were successful in achieving very good agreement with the experimental and simulation results of Baylor and Hollingworth [6] with respect to how average [Ca^2+^] changed over time after a simulated release of Ca^2+^. Our simulation also reinforces that there are very large localized differences in [Ca^2+^] that exist within the sarcomere [2, 5, 6], and that these differences exist for relatively long times, during a single activation. Also, the flexible nature of this model, and the ability to alter physical structures of the myofibril, such as the triad, SR, or MFL, will allow further exploration into the numerous disease states that alter these structures. Compared with previous simulations, our simulation introduces several novel features: the inclusion of the myofilament lattice, the actual simulation of the diffusion and reaction of Ca^2+^ throughout the sarcomere, and the ability to produce 3-D visualizations of the process, a potentially valuable pedagogical tool.

In this study we have demonstrated that the inclusion of the MFL changes the internal volumes within the simulation, and changes how Ca^2+^ ions are distributed within the sarcomere during a single activation. This change in distribution of Ca^2+^ results in more Ca^2+^ being distributed in the compartments closest to the Z-disk, and a corresponding decrease in the distribution of Ca^2+^ towards the M-line. This change in the distribution of Ca^2+^ is also reflected in the way Ca^2+^ binds to the regulator protein, TnC, so that more TnC is bound with Ca^2+^ towards the Z-disk. While we were unable to include these stabilizing protein titin in the model at this time, it should be noted that titin has been demonstrated to interact with Ca^2+^ and is exposed in the sarcomere between the ends of the myosin filaments and the Z-disks. While we did not model exposed titin in our model, our construction of the thick filament is based on experimental measurements, as a result the volume of our modelled myosin includes the volume of titin interwoven in the thick filament. The additional benefit of this type of model, is the ability to create 3D visualizations or animations of the model at any time point of the simulation from any angle. This ability coupled with the ability to choose what processes or reactions will be visualized, gives the observer an almost unlimited number of ways to explore the data set. As a result, this type of modeling provides new insight into how Ca^2+^ diffuses during activation.

While our simulation simplifies both the complexity and number of reactions calculated by the simulation of Baylor and Hollingworth [6], it replicates the averaged experimental and simulation results well for a single activation. It is important to emphasize that our simulation was able to do this by modeling the diffusion of Ca^2+^ and reactions within the sarcomere in an entirely novel way. As computational power continues to develop, adding more complexity to this simulation model will be relatively straightforward. At this time though, our simulation model provides a good first step in this journey.

We see our structural simulation model as an important step to understand how the complex micro-architecture of the sarcomere affects the diffusion, binding and uptake of Ca^2+^ during a single activation. In the future, we will advance this model to explore how changes in sarcomere length affect the diffusion and binding of Ca^2+^ as the myofilament overlap and inter-filament spacing change with sarcomere length.

## Methodology

Our model of the sarcomere was developed in the open source software Blender 3D. The protein structures used in the model were based on protein crystal structures downloaded from the RCSB-Protein Data Bank, a web based repository of protein structures developed by Breman and colleagues [23] which is available at https://rcsb.org. All simulations were then run using MCell software which is detailed in papers by Stiles and Kerr [7, 17, 30]. MCell development is supported by the NIGMS- funded (P41GM103712) National Center for Multiscale Modeling of Biological Systems (MMBioS) and available at: https://mcell.org. Current versions of MCell are distributed along with a software toolkit called Cellblender that integrates into the 3D modeling software Blender 3D. The Blender 3D software is freely available from the website: https://blender.org. The Cellblender toolkit facilitates rapid development of 3D models of the nano-scale cellular environment. Detailed examples and tutorials on building cellular mesh models using Cellblender in Blender, for use in MCell are described in a paper by Czech and colleagues [18].

### Sarcomere model

Within MCell, protein structures, membranes, and organelles are described by a series of triangulated surface meshes, which represent the nano-scale environment in three dimensions. Complete and detailed descriptions of the MCell simulation algorithms are described in papers by Stiles and Kerr [7, 30].

As in the simulations of Cannel and Allen [5] and Baylor and Hollingworth [2, 3, 6], our simulation is based on a myofibril with a length of half a sarcomere. Due to the structural complexity of our model, and computational limitations, we further divided our myofibril model into a 1/8th circumference slice of the half sarcomere (Fig 1). The dimensions used in constructing the model are listed in Table 3. The interior faces of the myofibril model are defined as reflective surfaces, with the general assumption that the number of exiting ions would balance with the number of ions that would enter. The mammalian (represented by mouse EDL) synthetic sarcomere was modelled to represent a whole sarcomere length of 2.3 μm; at this length, the actin and myosin filaments were optimally overlapped (our actin filament was modelled at a length of 1.06 μm which is slightly shorter than the normal actin length of mouse EDL 1.16 μm [31] which has shifted our force length optimal plateau to a shorter length).

**Table 3:** Proteins and MC size used within the simulation.

Object	Length	Radius	Volume	No.
Half sarcomere	1.15 μm	500 nm	1.11E-1 μm^3^	1
Myosin half filaments	812 nm	9 nm	2.90E-4 μm^3^	71
Actin filaments	1.06 μm	3.3 nm	1.85E-5 μm^3^	120
MC-c0	228 nm	160 nm	2.04E-3 μm^3^	5
MC-m0	228 nm	160 nm	7.27E-3 μm^3^	5
MC-o0	228 nm	160 nm	1.25E-2 μm^3^	5

### Reactions and rates

The modelled reactions that could occur in the simulation and rates that governed them are listed in Table 4.

**Table 4:** Reactions and rates used in the sarcomere simulation.

Reactants	Products	Rate M^-1^s^-1^	Rate s^-1^
Ca^2+^+TnC	TnC-Ca^2+^	1.77E+08	1.54E+03
Ca^2+^+TnC-Ca^2+^	TnC-2Ca^2+^	8.85E+07	17.1
Ca^2+^+SERCA	SERCA-Ca^2+^	1.74E+08	6.97
Ca^2+^+SERCA-Ca^2+^	SERCA-2Ca^2+^	1.74E+08	8.71
SERCA-2Ca^2+^	SERCA	3.48	
ATP+Ca^2+^	ATP- Ca^2+^	1.36E+07	3.0E+04

### Measurement compartments

We subdivided the interior of the model into 15 measurement compartments (MCs). These MCs were transparent to the molecules and ions within the simulation, but enabled the measurement of molecules, ions, and reactions that occurred within their boundaries. Unlike the previous simulations of Cannell and Allen [5] and Baylor and Hollingworth [2, 3, 6], in which all the compartments maintained the same volume, we divided our sarcomere model evenly so that each MC was 1/5th the length and 1/3rd the radius of the sarcomere model (Fig 1). The resulting concentric radial slices were referred to as: center (MC-c), middle (MC-m), and outer (MC-o). The compartments were then numbered sequentially from the Z-disk, beginning with 0 and ending with 4 closest to the M-line. In contrast to previous simulations, where concentration of Ca^2+^ is assumed to be uniform throughout a compartment, our model allowed a continuous concentration gradient across any MC. Diffusion was simulated independent of the boundaries of the MCs. The number of ions within the measurement compartments at each time step and the volume of the respective compartment were used to calculate concentration.

### Protein models

A segment of the actin filament protein model, number 2W49 by Wu [24], was used to develop our actin filament mesh model. Then the classic myosin S1 head protein model, 2MYS by Rayment [22], was used as a mesh model for the myosin S1 heads on the myosin filament. There are two issues with using protein models from the RCSB-Protein Data Bank directly: the models needed to be converted into suitable mesh models for use in Blender and Mcell, and the complexity of the mesh models needed to be reduced to provide results within the maximum compute wall time of 720 hours. The final simplified protein mesh models used in our simulation maintained the volume, and shape of the original models from the RCSB-PDB, but sacrificed surface detail for compute speed.

### Myosin filament

Once we had simplified, scaled, mesh models for the myosin S1 heads, we modelled the myosin filament backbone as a simple cylinder 800 nm in length (half of a normal myosin filament) with a radius of 9 nm. The myosin S1 heads were then paired and placed along the cylinder according to published descriptions by Zoghbi [25] and AL-Khayat [26] to create models of the myosin filament. The distal 725 nm of myosin filament (projecting towards the Z-disk) contained S1 heads, whereas the final length (75 nm) connecting myosin to the M-line was the myosin bare zone [32]. Once this was done, the individual heads were merged with the cylinder to make one unified mesh model of the myosin filament. Myosin filaments that straddled the 1/8th dividing edge of the myofibril were sliced in half so that they would fit within the model space.

### Actin filament

Developing a model of the actin filament presented a greater challenge. The imported mesh model from the protein databank (2W49) included numerous mesh faces that were internal to the model. To eliminate this superfluous structural complexity, we created a simple cylinder of similar length and diameter (38.5 nm and 6.6 nm respectively), then deformed the mesh cylinder so that it closely matched 2W49. The newly modelled actin segment was 99.8% of the volume of 2W49 by Wu [24]. Along this newly built actin segement, we selected faces on the model where the TnC were located in 2W49, and defined these surfaces as TnC placement locations. We then defined these locations in a series of surface region files and told MCell to place individual TnC proteins at each of these sites during runtime. This was an important step, as it over-rides the default random placement algorithm in MCell. The individual 38.5 nm sections were then merged end to end, to develop the full actin filament length.

### Myofilament lattice

The MFL within the myofibril is dictated by how the actin and myosin filaments are positioned relative to each other. The myofibril is isovolumetric [10], so the spacing between the filaments increases and decreases as the sarcomere shortens and lengthens, respectively. To determine the correct filament spacing for our model at a length of 2.3 μm, we used the equation developed by Millman [10] that relates sarcomere length (SL) with lattice spacing (LS) (Eq 1). Using Eq 1, and assuming a lattice volume (LV) for mouse muscle of 4 × 10^-3^ μm^3^ [33], we calculated the correct myofilament lattice spacing for a SL of 2.3 μm and incorporated it into our model (Eq 1).

### Sarcoplasmic reticulum

The SR model is based on the descriptions and images presented by Ogata [13]. The high resolution images of the SR from Ogata [13] were wrapped around the outside surface of our myofibril model and used as a template to define the SR. This part of the model is a defined surface, populated with SERCA pumps. The triad complex was positioned 500 nm from the Z-disk, as described by Brown [20] and Gomez [29] with the terminal cisternae on either side of the T-tubule. RYR placement was confined to the surface designated as terminal cisternae (Fig 7). At runtime, MCell positioned SERCA pumps and RYR release sites randomly across their defined surfaces until the determined density was reached (Fig 7).

### SERCA pumps

SERCA pumps were placed at a concentration of 240 μM, as described by Fanzini-Armstrong [19] and Baylor and Hollingworth [6], although only 60% of them were allowed to be active. Activity of RYR was assigned pseudo-randomly at runtime based on seed number. The reason for using this activity percentage was that our model did not include Mg^2+^ ions, and we could not use the more advanced reaction equations used by Baylor and Hollingworth [6] that simulate the competitive binding of the SERCA pump by Ca^2+^ and Mg^2+^.

### Simulated action potential

For computational efficiency, we simulated Ca^2+^-release from the RYR by creating a reaction rate file, which dictated the rate at which Ca^2+^ were generated from the RYR. The values in the reaction rate file were changed every 100 microseconds to allow Ca^2+^ release to mimic the release equation used by Baylor and Hollingworth [6] (Fig 2). To determine if this was successful, the release rates were plotted and integrated over time. From this plot the peak release rate, the full duration at half max (FDHM), and total amount of Ca^2+^ released was determined. These data were compared with the data of Baylor and Hollingworth [6].

### Software and computer specifications

The cellular micro-architecture was built using Cell-Blender tools version RC 1.0, in Blender 2.68b. Monte Carlo simulations were performed using MCell version 3.2.1, and simulation runs were carried out on a variety of computer hardware. Initial development was completed on a 64bit Debian GNU/linux machine, running on an Intel iCore-7-3770k processor with 24 Gb of RAM. Repeated trials with different seed values were computed in parallel, on the Compute Canada Resources computer clusters Storm and Breeze, in the WestGrid resource pool (www.computecanada.ca). The resulting complexity of the model required us to select a simulation step size of 0.6 ns to minimize the probability of missing any reaction. Reaction data were recorded every 60 ns. Simulations were run for 11.66E+6 steps to provide 7 ms worth of data. As the model is stochastic, 10 simulations with seed numbers 1-10 were run. One simulation run (seed 10) was continued for 23.32E+6 steps to simulate 14 ms, and one visualization run (seed 9) was completed where the position and state of all model elements were recorded every 1 μs. For data safety, a data checkpoint file was created every 6 hours so that in the event of a computer failure or shutdown, the simulation could be restarted beginning at the last 6-hour interval. The results from seed 9 were used to create the animations and 3D visualizations. The visualizations record 1 frame of the model every 5000 iterations, so the final animation takes a reasonable time to view. This does create the illusion in the animation that the ions take much larger steps in their random walk than they actually do.

Raw data files from the results were processed and analyzed with Matlab-14a using a series of custom developed functions to consolidate the large data files. Matlab was used to plot the results, and compile general statistics such as standard deviations and means. Means and standard deviations were calculated for the whole model as well as the MCs within (S1 Video).

Paired T-tests were used to compare pairs of MCs within the model to determine how [Ca^2+^], peak number of Ca^2+^ ions, and the time at which peak [Ca^2+^] occurred. Alpha was set at 0.01. The Paired T-test was used as one compartment was directly compared with another compartment; only two comparisons were made.

## Supporting information

S1 Fig Perspective rendering of myofilament lattice. The myofilament lattice and structure of the model is rendered from the perspective of a calcium ion (looking directly down the filament towards the m-line) within the simulation. The myosin filaments with attached S1 heads are blue, and the actin filaments are red. ATP molecules are yellow and are represented at 50 times larger than their actual size in the simulation (which can lead to overlapping of the meshes), so they are easy to see within the simulation.
(TIF)

S2 Fig Second perspective rendering of myofilament lattice. The myofilament lattice and structure of the model is rendered from within the simulation looking across the MFL from a 30 degree angle. The myosin filaments with attached S1 heads are blue, and the actin filaments are mostly red with potential myosin binding locations in green. ATP molecules are yellow and are represented at 50 times larger than their actual size in the simulation.
(TIF)

S1 Video Animation of half sarcomere model highlighting Ca^2+^ release, and diffusion. The diffusion and buffering action of ATP is also demonstrated. Data from seed 09 with camera view rotating around the model. The diffusion and binding of Ca^2+^ is highlighted alone with the diffusion and buffering action of ATP. All myofilaments are present in the model but only one actin and myosin filament are shown so as not to obscure the visibility of Ca^2+^.
(MP4)

S2 Video Animation of half sarcomere model highlighting the binding of Ca^2+^ to Tn-c locations. All the myofilaments are present in the simulation although only one actin and myosin filament are rendered. This helps to see orientation but not obscure the Tn-C binding locations.
(MP4)
