# Supplementary figures and images for: A stochastic simulation of skeletal muscle calcium transients in a structurally realistic sarcomere model using MCell
Source: PLoS Comput Biol. 2019 Mar 7;15(3):e1006712. doi: 10.1371/journal.pcbi.1006712 (PMC6424466; doi:10.1371/journal.pcbi.1006712)

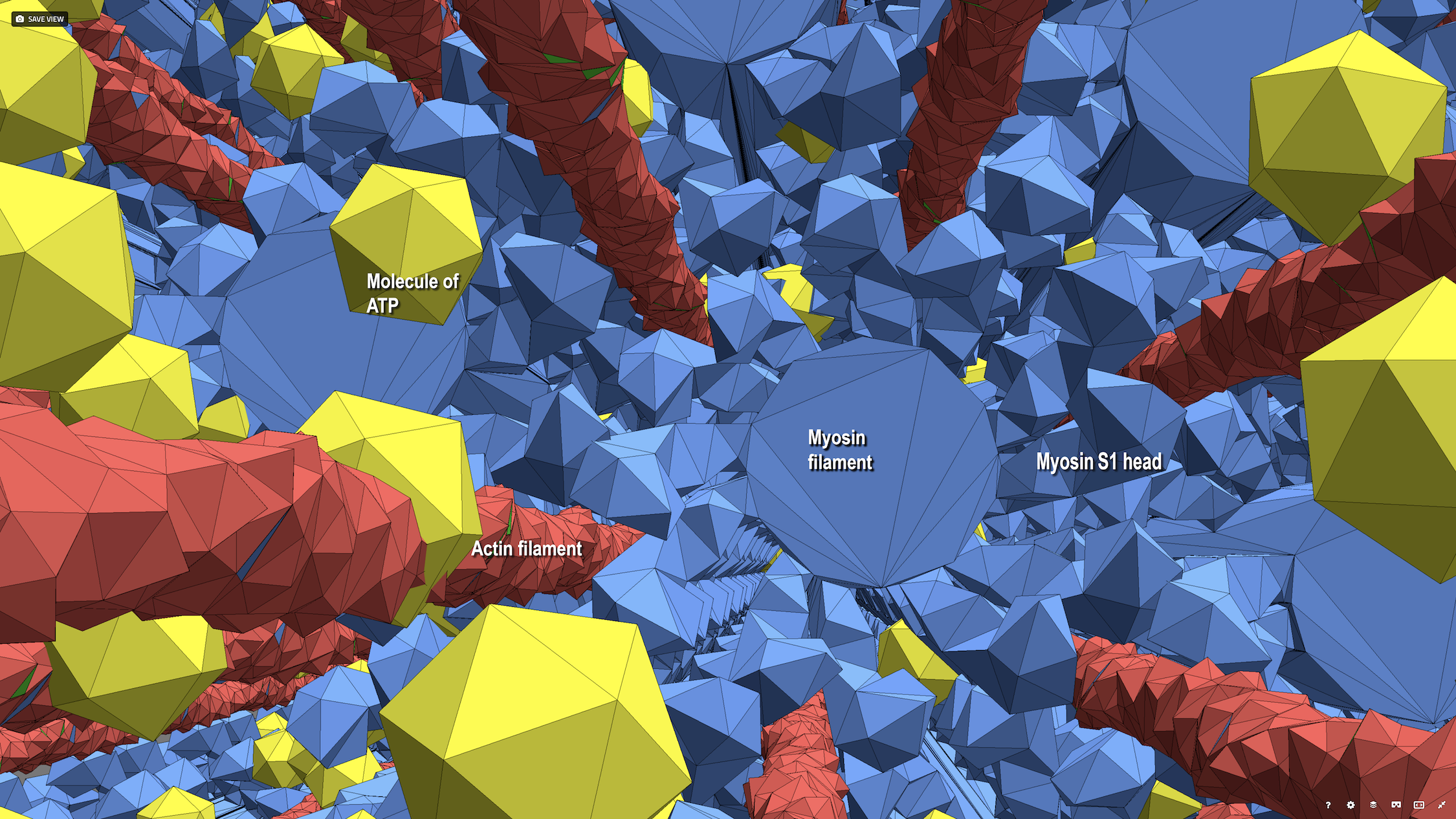

Supplement: S1 Fig — The myofilament lattice and structure of the model is rendered from the perspective of a calcium ion (looking directly down the filament towards the m-line) within the simulation. The myosin filaments with attached S1 heads are blue, and the actin filaments are red. ATP molecules are yellow and are represented at 50 times larger than their actual size in the simulation (which can lead to overlapping of the meshes), so they are easy to see within the simulation. (TIF) [file pcbi.1006712.s001.tif]

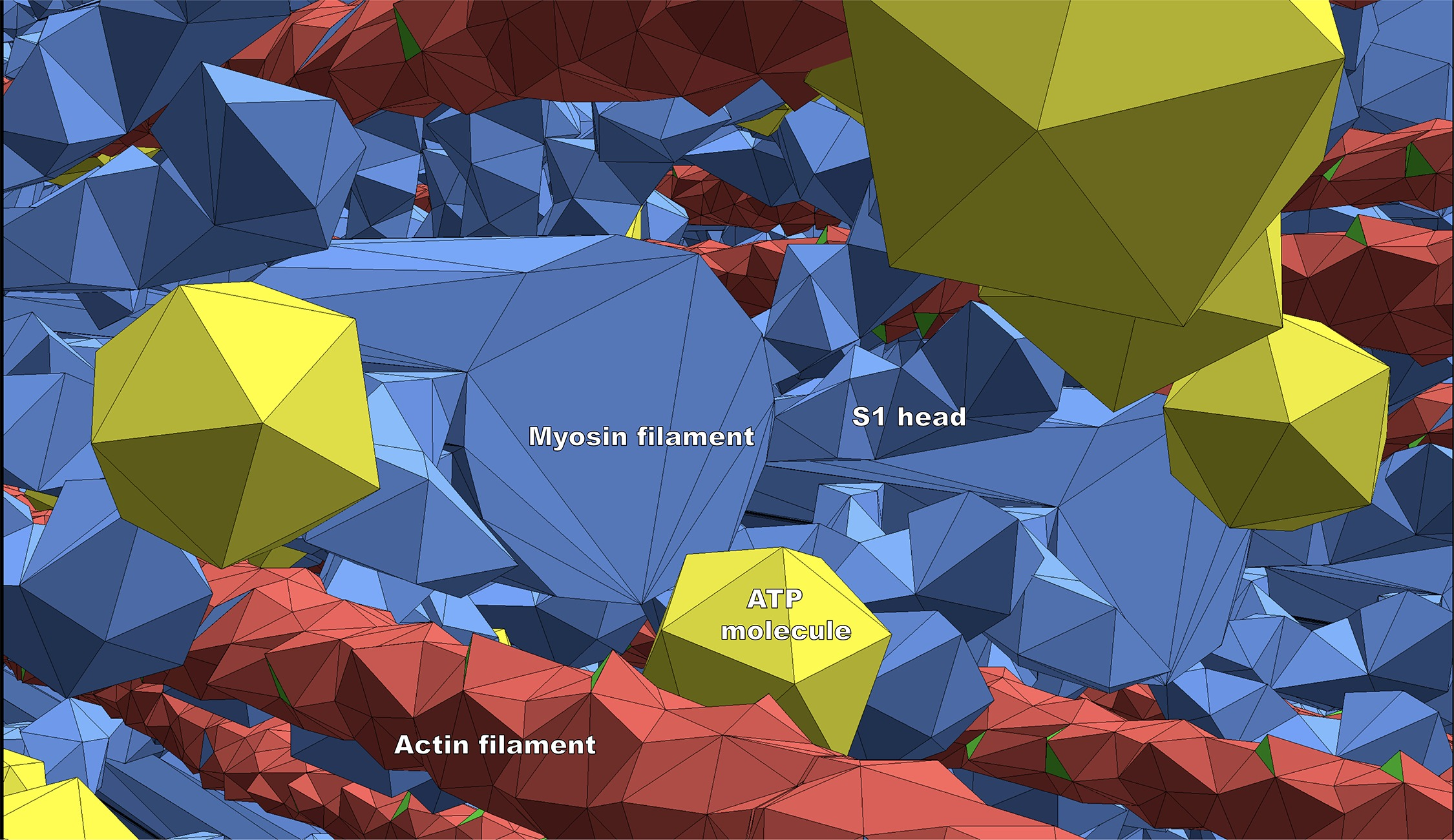

Supplement: S2 Fig — The myofilament lattice and structure of the model is rendered from within the simulation looking across the MFL from a 30 degree angle. The myosin filaments with attached S1 heads are blue, and the actin filaments are mostly red with potential myosin binding locations in green. ATP molecules are yellow and are represented at 50 times larger than their actual size in the simulation. (TIF) [file pcbi.1006712.s002.tif]
